# Introduction to semantic e-Science in biomedicine

**DOI:** 10.1186/1471-2105-8-S3-S1

**Published:** 2007-05-09

**Authors:** Huajun Chen, Yimin Wang, Zhaohui Wu

**Affiliations:** 1 College of Computer Science, Zhejiang University, Hangzhou, 310027, P.R. China; 2 Institute AIFB, University of Karlsruhe (TH) Karlsruhe, 76128, Germany; 3 Yale Center for Medical Informatics, Yale University, New Haven, 06511, USA

## Abstract

The Semantic Web technologies provide enhanced capabilities that allow data and the meaning of the data to be shared and reused across application, enterprise, and community boundaries, better enabling integrative research and more effective knowledge discovery. This special issue is intended to give an introduction of the state-of-the-art of Semantic Web technologies and describe how such technologies would be used to build the e-Science infrastructure for biomedical communities. Six papers have been selected and included, featuring different approaches and experiences in a variety of biomedical domains.

## Introduction

Advances in biotechnology and computing technology have made the information growth in biomedicine phenomenal. With the exponential growth in complexity and scope of modern biomedical research, it is becoming more and more urgent to support wide-scale and ad-hoc collaboration and exchanging ideas, information and knowledge across organizational, governance, socio-cultural, and disciplinary boundaries. Researchers working on one aspect of analysis may need to look for and explore results from other institutions, from other subfields within his or her discipline, or even from completely different biomedical disciplines. For example, the research on neuron-related diseases such as Parkinson, Alzheimer, Huntington, require the researcher to combine knowledge from different research institutions and spans the disciplines of neuroscience, psychiatry, biochemistry, molecular biology, computer science, and so forth. These kinds of requirements are commonly known as "e-Science", which is scientific investigation performed through distributed global collaborations between scientists and their resources, and the informatics infrastructure that enables this.

Current Web technologies fall short in terms of fulfilling the requirements of advanced e-Science. A new breed of Web technologies has been emerging with the potential to change the way and enhance the ability of scientists to do research. Among these technologies, the Semantic Web [1], initiated and promoted by W3C [2], is aimed to provide enhanced capabilities that allow data and the meaning of the data to be shared and reused across application, enterprise, and community boundaries, by explicitly encoding data semantics as machine-understandable vocabularies, thesauri, and ontologies.

To achieve this goal, the Semantic Web community has proposed and developed new standard Web languages such as RDF (the Resource Description Framework) [3] and OWL (the Web Ontology Language) [4], which provide enhanced capability for resource description and knowledge representation going far beyond the content presentation capabilities of HTML language and data tagging capabilities of the XML language. These languages can be used to represent meaning of data, define metadata, specify terminologies and vocabularies, describe the input/output and functional capability of web service, and so forth. By focusing on the semantics of information, more intelligent system can be built to allow logic-based query and semantic-based data reasoning across an probably unbounded web of linked data repositories, better enabling effective knowledge discovery and integrated research across organization boundaries [5-10].

The potential of the Semantic Web technologies has been recognized in the health care and life science communities. For example, the World Wide Web Consortium (W3C) has established a Semantic Web interest group to focus on Health Care and Life Sciences (HCLSIG) [11]. We have observed a steady increase of efforts in this new direction. For examples, several major biomedical ontology efforts have been launched lately: OBI [12] project is developing an integrated ontology for the description of biological and medical experiments and investigations; the RNA Ontology Consortium (ROC) is created to develop an RNA Ontology (RO) [13]. We have also witnessed advocate of applications in neuroscience [14], drug discovery [15,16], public health surveillance [17], knowledge discovery application [18], and scientific publishing [19].

This special issue is intended to give an introduction of the state-of-the-art Semantic Web technologies and describe how such technologies would be used to build the e-Science infrastructure for biomedical communities. Six papers have been selected and included, featuring different approaches and experiences in a variety of biomedical domains.

## Special issue summary

### Overview

In this special issue, there is one community paper [20] which was authored by members of the W3C's healthcare and life science interest group, envisioning the future application of semantic web technologies in translational medicine research; three research papers from the perspectives of semantic modelling of biomedicine knowledge [21], ontology-based categorization of human disease genes [22], and RDF-based management of graph of identifier relationships of biomedical resources [23]; and two application papers introducing the application of semantic technologies in neuroscience [24] and traditional Chinese medicine communities [25].

### Translational medicine research with semantic web

The authors envision the use of Semantic Web technologies will improve the productivity of translational research, accelerate the movement of discoveries in basic research (the Bench) to application at the clinical level (the Bedside). It introduces the current efforts of the W3C's HCLSIG. The paper also presents a scenario that shows the value of the information environment the Semantic Web can support for aiding neuroscience researchers, and reports on several projects by members of the HCLSIG.

### Modelling biological pathway with semantics

The authors report the BioPAX [26] which is a collaborative effort to create a data exchange format for biological pathway data. In this paper, the authors explore the potential for the BioPAX initiative and the BioPAX ontology to model and deliver the pathway data necessary for systems style bioinformatics. The authors demonstrate how to map different pathway database to the BioPAX ontology, and the way of using the BioPAX ontology to ask questions over different pathway databases.

### Neuroscience meets semantic e-Science

The authors present a semantic web approach to building an e-Neuroscience framework by using RDF(S) language. They have constructed an ontology for BrainPharm of Yale SenseLab using RDFS and then converted a subset of the BrainPharm data into RDF according to the ontological structure. They have also integrated the converted BrainPharm data with the hypothesis and publication data in RDF from a SWAN[27] (Semantic Web Applications in Neuromedicine) pilot version. Their implementation makes use of Oracle RDF Data Model for data integration, query, and inference.

### LinkHub

The authors report a software system called LinkHub using RDF to manage the relationship graph of biomedical resource identifier and allow exploration with a variety of interfaces. LinkHub also facilitates queries and access to information and documents related to identifiers spread across multiple databases, acting as "connecting glue" between different identifier spaces. The authors have used LinkHub to establish such a relationship between UniProt and the North East Structural Genomics Consortium.

### Semantic e-Science for traditional Chinese medicine

The authors present the entire vision and current status of applying semantic web and knowledge-based techniques in Traditional Chinese Medicine (TCM) based on semantic. The authors use domain ontologies to integrate TCM database resources and services in a semantic cyberspace and deliver a semantically superior experience including browsing, searching, querying and knowledge discovery to users.

### Functional categorization of human disease genes using gene ontology

The authors evaluate automated classifications of human disease genes using their Gene Ontology annotations. Two automated methods are proposed to investigate the classification of human disease genes into independently predefined categories of protein function. One method uses the structure of Gene Ontology by preselecting 74 Gene Ontology terms assigned to 11 protein function categories. The second method is based on the similarity of human disease genes clustered according to the information-theoretic distance of their Gene Ontology annotations.

## Conclusion

e-Science has entered into an era, in which, scientific discovery will increasingly rely on networked information environment and integrated information resources. With the growing number and diversity of digital resources in biomedicine, we can look forward to a promising future for Semantic Web technologies in this field.

Challenges and obstacles, however, remain ahead of the path. Ontology is useful for data integration and knowledge sharing across organization boundaries, but providing agreed ontological structures to legacy databases can be very time-consuming and labor-intensive. This should be done in an incremental fashion. While rich semantics is necessary, a little semantics really goes a long way.

As pointed out by Benjamin M. Good [28], like the original Web, the establishment of the life science Semantic Web will depend primarily on the will and participation of its consumers. We expect this special issue would not only provide valuable application experiences for Semantic Web researchers and developers, but also help health care and life science researchers to better understand the potential benefits and values Semantic Web technologies could bring to their daily research endeavors.

## Competing interests

The authors declare that they have no competing interests.
